# Development of optical choppers for time-resolved measurements at soft X-ray synchrotron radiation beamlines

**DOI:** 10.1107/S1600577517002399

**Published:** 2017-03-20

**Authors:** Hitoshi Osawa, Takuo Ohkochi, Masami Fujisawa, Shigeru Kimura, Toyohiko Kinoshita

**Affiliations:** a Research and Utilization Division, Japan Synchrotron Radiation Research Institute (JASRI), Kouto 1-1-1, Sayo, Hyogo 679-5198, Japan; b Synchrotron Radiation Laboratory, Institute for Solid State Physics, University of Tokyo, Kashiwanoha 5-1-5, Kashiwa, Chiba 277-0882, Japan

**Keywords:** X-ray chopper, soft X-ray, time-resolved measurements, ultra-high vacuum

## Abstract

Two types of optical choppers for time-resolved measurements at synchrotron radiation soft X-ray beamlines have been developed. One type uses an air-spindle-type rotation mechanism with a two-stage differential pumping system to maintain the ultra-high vacuum of the X-ray beamline, and the other uses a magnetic bearing. Both can be installed at the soft X-ray beamlines at SPring-8, greatly improving the accessibility of pump-and-probe spectroscopy. The combination of X-ray chopper and pump-and-probe photoemission electron microscope at SPring-8 provides drastic improvements in signal-to-noise ratio and resolution compared with techniques using high-voltage gating of channel plate detectors. The choppers have the capability to be used not only at synchrotron radiation facilities but also at other types of soft X-ray and VUV beamlines.

## Abstract 2

Two types of optical choppers for time-resolved measurements in soft X-ray beamlines of synchrotron radiation facilities were developed, which are utilized under ultra-high-vacuum conditions.

## 1. Introduction   

Pump-and-probe time-resolved measurements using pulsed light are a recent trend in a variety of scientific fields, and ultra-fast measurements using laser pulses can now access the attosecond timescale region. Although the duration of synchrotron light pulses is usually dozens of picoseconds, much longer than laser pulses, many interesting and important subjects can be investigated dynamically (Fukumoto *et al.*, 2008 ▸; Kinoshita *et al.*, 2012 ▸; Ohkochi *et al.*, 2012 ▸; Yamamoto & Matsuda, 2013 ▸; Neppl & Gessner, 2015 ▸). The tunability of the photon energy of synchrotron radiation (SR) is a big advantage, especially in spectroscopic studies. However, for pump-and-probe time-resolved measurements using SR, it is necessary to select individual X-ray pulses (bunches), because the repetition frequency of the ‘probe’ SR pulses is usually much higher than that of the ‘pump’ laser pulses used to excite the sample. At SPring-8, some different bunch modes (see http://www.spring8.or.jp/en/users/operation_status/schedule/bunch_mode_b16/) are available during user beam time. Currently there is no provision of a single-bunch mode, since the reduced average photon flux would impact the experiments of users not performing time-resolved measurements. There are, however, several ways to make single-bunch measurements using the various bunch modes provided at SPring-8. One is to construct gating circuits for the pulse-counting system, as used in previous works (Fukumoto *et al.*, 2008 ▸; Kinoshita *et al.*, 2012 ▸; Ohkochi *et al.*, 2012 ▸), and another is to use a detector system with sufficient time-resolution (Yamamoto & Matsuda, 2013 ▸). Another approach is to select single X-ray pulses using X-ray choppers, as is done at many SR facilities (Lindenau *et al.*, 2004 ▸; Cammarata *et al.*, 2009 ▸; Gembicky & Coppens, 2007 ▸; McPherson *et al.*, 2000 ▸, 2002 ▸; Meents *et al.*, 2009 ▸).

Recently, a high-repetition-rate X-ray chopper which can operate at over 50 kHz has been developed at SPring-8 (Osawa *et al.*, 2017 ▸). The apparatus has been installed at a number of beamlines, and successfully operated during user beam time. In contrast to choppers operating in the hard X-ray region, there have been fewer reports of experiments using choppers in the soft X-ray region, since soft X-ray choppers must be compatible with the ultra-high vacuum of a soft X-ray beamline. In order to overcome this difficulty, we have developed two types of soft X-ray chopper. One uses an air-spindle-type rotation mechanism with a two-stage differential pumping system to maintain ultra-high-vacuum conditions, and the other uses a magnetic bearing. In the present paper, the detailed designs and performance of these two chopper systems are introduced.

## 2. Instrumentation   

### 2.1. Design concept of the soft X-ray choppers   

**Figure 1:**
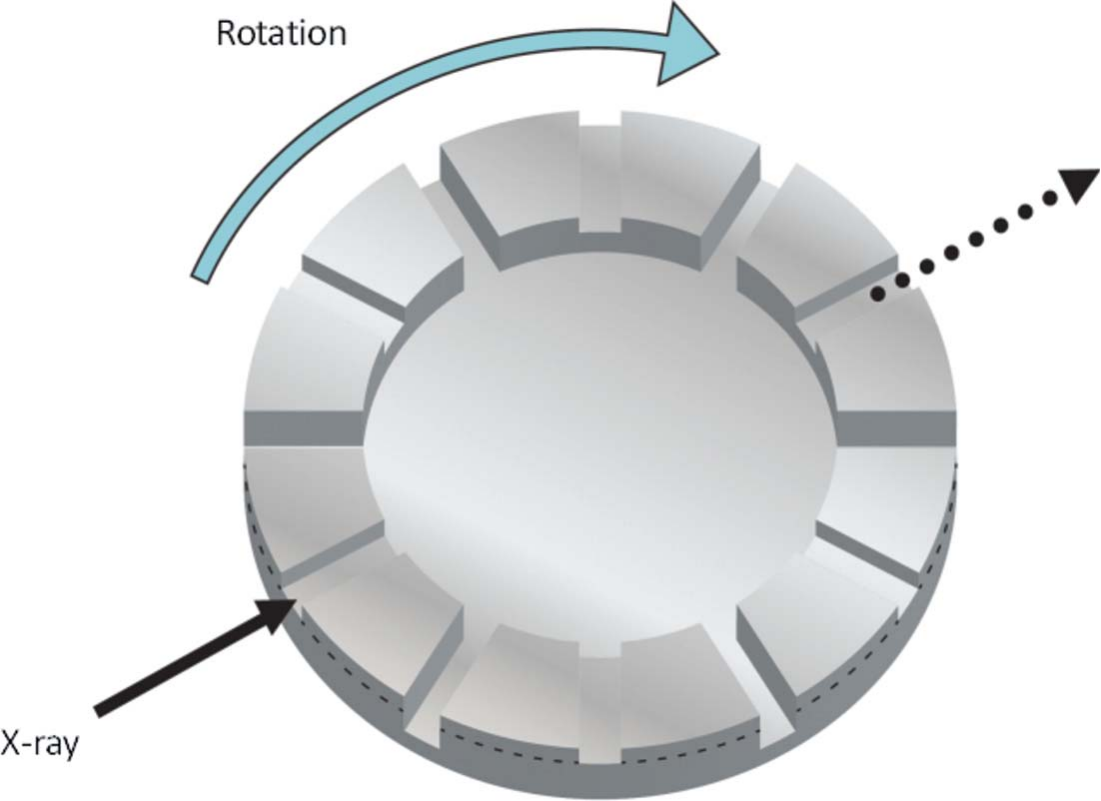
Schematic view of the X-ray chopper design used at SPring-8. Two different groove depths are machined, allowing two different repetition frequencies to be selected by aligning the X-ray beam axis above or below the plane defined by the broken line shown. Eight pairs for the air-spindle type and five and ten pairs for the magnetic-bearing type are grooved.

The design concept of the soft X-ray choppers is similar to that of the hard X-ray choppers reported previously (Osawa *et al.*, 2017 ▸). The axis of rotation of the chopper is perpendicular to the X-ray beam, offering the advantage of short opening times. The chopper shape is a disc, and the X-rays pass through grooves cut along radial lines on the surface of the disc, as shown in Fig. 1 ▸, where a design which can operate at two different repetition frequencies is shown. In this design, grooves with two different depths are machined into the disc. By changing the position of the grooves relative to the X-ray axis (above or below the broken line in the figure), different repetition frequencies can be selected. This grooved design is of low fabrication cost, and is well suited to high-chopping-frequency applications which require large numbers of grooves. Compared with choppers designed for the hard X-ray region, the grooves can be much shorter due to the shorter absorption length in the disc material at lower photon energies. We use a Duralumin disc for the air-spindle-type chopper and a Ti disc for the magnetic-bearing-type chopper. The latter design rotates at a higher speed, so a tougher material is required to prevent deformation due to centrifugal forces.

As for the previous hard X-ray version, the speed and phase of the motor are controlled by a phase-locked loop linking the SPring-8 RF pulse to the eight pulses per revolution required to control the motor, developed by ShinMaywa Inc.

The SPring-8 storage ring is operated with a variety of bunch modes to cater for different experimental techniques (see http://www.spring8.or.jp/en/users/operation_status/schedule/bunch_mode_b16/). Modes suitable for pump-and-probe measurements are modes A (203 bunches with a 23.6 ns interval, 0.4–0.5 mA bunch^−1^), D (1/7-filling + 5 bunches with a 684.3 ns interval, 3 mA in each of the 5 single bunches), E (2/29-filling + 26 bunches with a 165.2 ns interval, 1.4 mA in each single bunch), F (1/14-filling + 12 bunches with 342 ns interval, 1.6 mA in each single bunch) and H (11/29-filling + 1 bunch with a 1486 ns interval either side, 5 mA for the single bunch). The present soft X-ray chopper is designed for selecting the isolated single bunch of the H-mode.

The train part of the H-mode filling pattern can also be used for time-resolved measurements. Here, 95 mA of storage current can be used as a long photon pulse of 1467 ns. This operation is suitable for investigating relatively slow phenomena, in the several microsecond to sub-millisecond range.

### 2.2. Air-spindle-type chopper apparatus   

As reported in the previous paper (Osawa *et al.*, 2017 ▸), the hard X-ray chopper developed at SPring-8 works quite well. However, this type of chopper is not suitable for soft X-ray beamlines, where it is necessary to maintain a vacuum of 10^−7^ Pa or lower. The air spindle rotation axis needs a high-pressure air flow to maintain stable rotational conditions without mechanical friction, leading to a large gas load. In order to maintain the ultrahigh vacuum of the beamline, the new chopper design uses a two-stage differential pumping mechanism installed in the rotation axes. The pumping and air flow paths are carefully designed to provide an efficient pumping effect.

**Figure 2:**
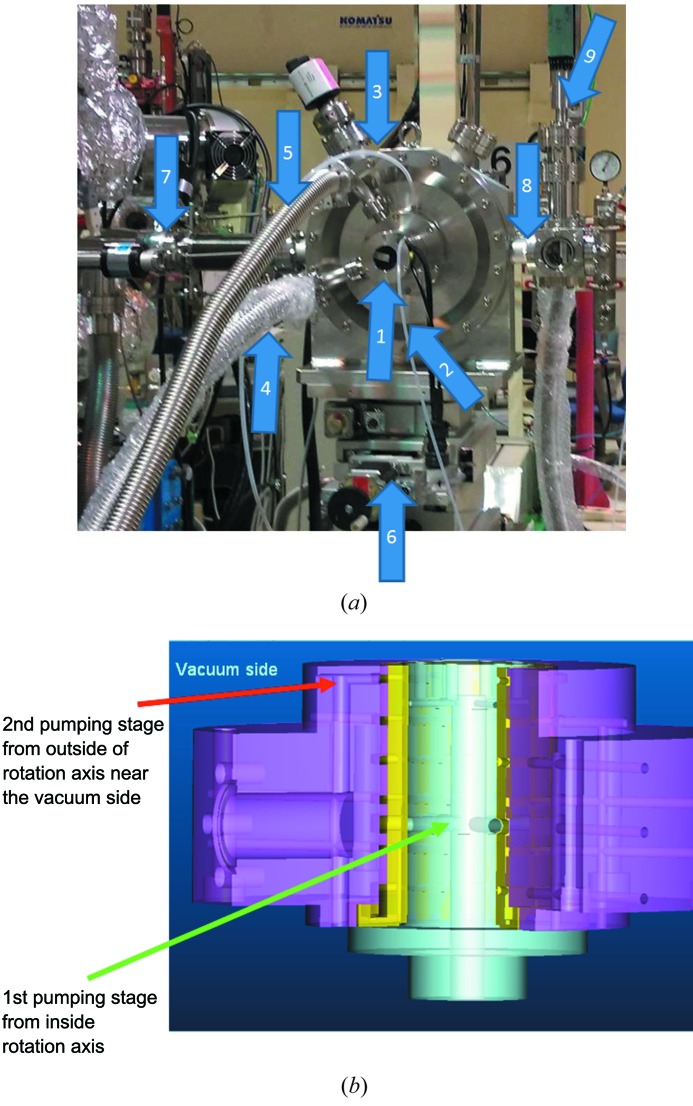
(*a*) Photograph of the air-spindle soft X-ray chopper apparatus. The X-ray beam passes from left (upstream) to right (downstream). (1) Rotation axis. (2) Air inlet for axis cooling. (3) Air inlet for spindle. (4) Pumping port for second stage [see (*b*), turbo-molecular pump]. (5) Pumping port for first stage [see (*b*), scroll pump]. (6) *X–Y*–θ stage. (7) Upstream port, with 5 mm-diameter aperture. (8) Same as (7) but for downstream. (9) Port for PIN photodiode. (*b*) Cross-sectional view of the differential pumping system of the rotational axis. The part shown in light blue is the rotator, and that shown in yellow is the coated and polished wall for the air spindle.

The design and construction was performed by ShinMaywa Inc. The soft X-rays pass along the direction shown in Fig. 1 ▸ through grooves in the 190 mm-diameter disc, onto which eight pairs of grooves are machined. (Although six pairs are drawn in the figure schematically, one can machine a different numbers of pairs depending on the repetition frequency.) An overview of the apparatus is shown in Fig. 2(*a*) ▸. The system is mounted on an *X–Z*–θ stage, which is used for precise alignment of the X-ray axis. The rotation axis is horizontal, and the disc is mounted vertically. Air inlet tubes and two pumping ports are arranged around the rotational axis. Fig. 2(*b*) ▸ shows a cross-sectional view of the differential pumping system of the rotational axis. The top side of the figure corresponds to the vacuum side of the chamber. From the bottom, air is introduced at high pressure (>0.7 MPa). The first stage of differential pumping is carried out from the inside of the rotational axis, and the second stage consists of pumping from the outside of the axis, and is located much closer to the vacuum side. In addition to the differential pumping system, two apertures are inserted, upstream and downstream of the chopper chamber, as shown in Fig. 2(*a*) ▸. The diameter and length of the apertures are 5 mm and 11 mm, respectively, machined on ICF 70 flanges. This enables us to save space and create a large pressure gradient between the chopper and the other parts of the beamline. The rotation speed is ∼18000 rpm, and the aperture size (groove width × depth) is ∼450 µm × 1300 µm.

### 2.3. Magnetic-bearing-type apparatus   

The air-spindle-type rotation mechanism works well, as discussed below. However, problems with the differential pumping system or accidental losses of electric power could potentially lead to damage of the beamline due to the introduction of gas at atmospheric pressure. It is therefore not practical to permanently install the system into a soft X-ray beamline. In contrast, magnetic-bearing rotation systems are widely utilized under ultra-high-vacuum conditions, such as the rotation axes of turbomolecular pumps. The rotation speed of a magnetic-bearing system is also suitable for use in the current soft X-ray chopper, and multi-directional stabilizing systems are available.

**Figure 3:**
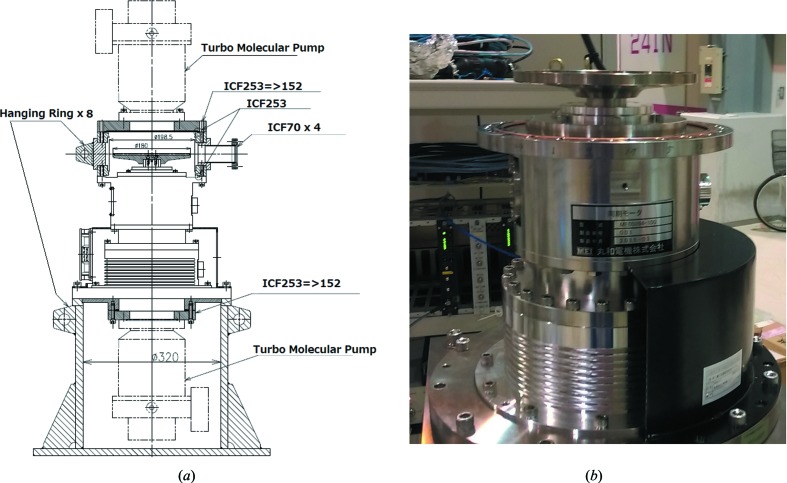
Schematic drawing (*a*) and photograph (*b*) of the magnetic-bearing chopper. Dimensions are given in mm.

The apparatus was designed and constructed by Maruwa Electric Inc. The diameter of the chopper disc is 180 mm. Two stabilizing systems are mounted on the top and bottom of the axis, and control stability in the *X–Y–Z* directions. Figs. 3(*a*) and 3(*b*) ▸ show a schematic drawing and photograph of the magnetic bearing chopper, respectively. In contrast to the air-spindle design, the disc rotates around the vertical axis, with higher speeds of up to ∼30000 rpm. Therefore, the opening width of the grooves is also wider, at ∼640 µm × 1500 µm and ∼640 µm × 3000 µm. As for the hard X-ray chopper, two stages of groove pairs (five pairs and ten pairs for 5 kHz and 10 kHz operations, respectively), corresponding to below and above the broken line in Fig. 1 ▸, are machined. The frequency is selected by changing the height of the *Z*-stage of the apparatus. The turbomolecular pumping system is located below the magnetic-bearing system.

## 3. Performance of the soft X-ray choppers   

The performances of the developed chopper systems were investigated at BL25SU (Senba *et al.*, 2016 ▸) of SPring-8. The soft X-ray chopper chamber was installed at the middle of the vacuum duct between the exit slit and the refocusing mirror chambers of Branch A of the beamline. The pressure was measured at the chopper chamber and several points of the beamline. We did not perform any baking procedures for either of the apparatuses. Under operation, the pressure for the air-spindle chopper reached 6 × 10^−5^ Pa; however, the pressures in the upstream and downstream beamline ducts remained lower than 2 × 10^−6^ Pa due to the two apertures described in §2.2. Thus the vacuums of the slit chamber and the refocusing mirror chambers were not affected. For the magnetic-bearing chopper, a pressure of 1 × 10^−6^ Pa was achieved.

**Figure 4:**
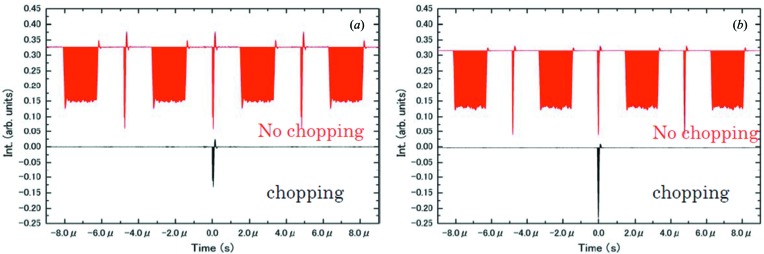
Time structure of X-ray pulses with and without chopping for the air-spindle chopper (*a*) and the magnetic-bearing chopper (*b*). These spectra were recorded during H-mode operation of SPring-8.

**Figure 5:**
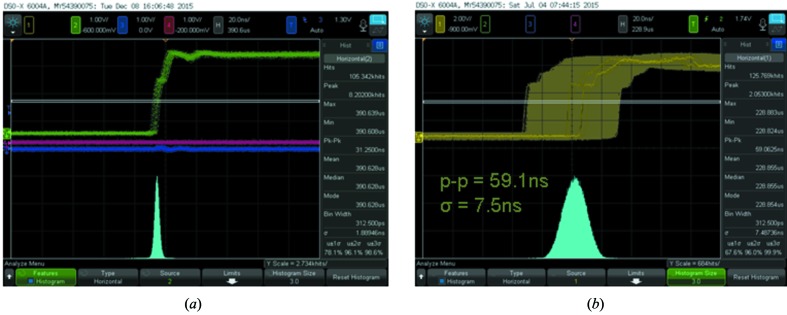
Observed signals from the rotation axis position encoders of the air-spindle (*a*) and the magnetic-bearing (*b*) choppers.

In order to check the time profile of photons downstream of the chopper, we measured the photocurrent using a PIN photodiode (Hamamatsu Photonics, S3584-09; reverse bias voltage 18–20 eV), using photon energies of 740 eV (air-spindle chopper) and 700 eV (magnetic-bearing chopper). Fig. 4 ▸ shows results obtained during H-mode operation. The time spectra were recorded using an oscilloscope. Fig. 5 ▸ shows the time structure of X-ray pulses without and with the chopper both for the air-spindle and for the magnetic-bearing types. The bunch structure of H-mode is clearly seen, *i.e.* one isolated bunch and a multi-bunch portion. It can be seen that for both types of chopper all pulses except the target pulse are effectively blocked. The throughput intensity of the target pulse is different, at 49% for the air-spindle-type and 80% for the magnetic-bearing type, due to the difference in groove width.

As reported previously (Osawa *et al.*, 2017 ▸), the motors for the rotation axes have rotary encoders. Fig. 5 ▸ shows the observed signals from the encoders as recorded by an oscilloscope for the air-spindle (*a*) and the magnetic-bearing (*b*) choppers, at chopper speeds of 18000 and 30000 rpm, respectively. From the figure, rotation jitters of 31.3 ns with a Gaussian width of 1.9 ns (FWHM) and 59.1 ns with a 7.5 ns width (FWHM) can be confirmed, which are sufficiently narrower than the intervals between the single bunch and the train portion of H-mode at SPring-8. The magnetic-bearing design is, however, susceptible to accidental vibrations from outside of the apparatus, which can lead to large jitters with recovery times of several seconds. For this reason, care must be taken with installation.

**Figure 6:**
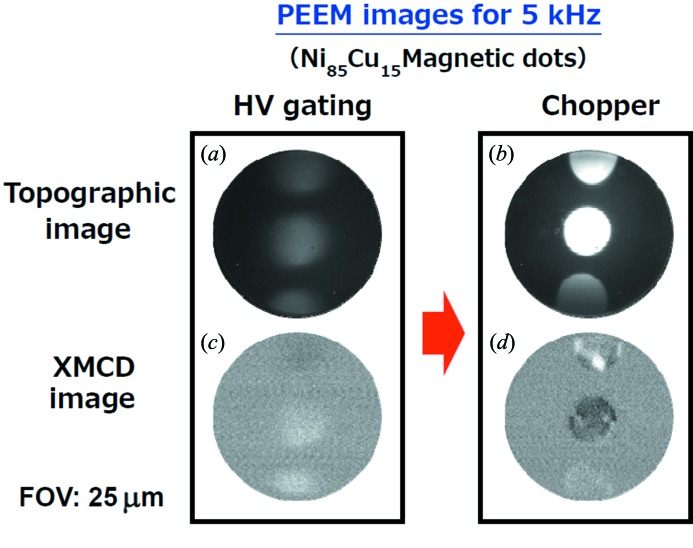
PEEM images of Ni_85_Cu_15_ magnetic dots. (*a*, *b*) Topographic images, and (*c*, *d*) XMCD-PEEM images, obtained by the electric gating and mechanical chopping methods. The images were taken at the Ni *L*-edge with a field of view of diameter 25 µm. The exposure time was 3.8 s × 18 cycles. The air-spindle-type chopper was used for taking images in (*b*) and (*d*).

Good performance of the chopper systems was also verified by observing photoemission electron microscopy (PEEM) images in the SR pulse selection mode. Previously reported pump-and-probe time-resolved PEEM studies at SPring-8 (Fukumoto *et al.*, 2008 ▸; Kinoshita *et al.*, 2012 ▸; Ohkochi *et al.*, 2012 ▸) used high-voltage pulses (∼600 V, 300 ns width) applied to the microchannel plate detector in order to selectively visualize only photoelectrons activated by single SR pulses, leading to effectively time-resolved measurements. However, the AC electrical noise due to the high-frequency pulsed voltages modulates the focusing bias of the lens system, which in turn deforms the focal condition of the images. Fig. 6 ▸ shows topographic and X-ray magnetic circular dichroism (XMCD) PEEM images of Ni_85_Cu_15_ dots (diameter 7 µm) obtained at the *L*_2,3_-edge of Ni. For comparison, the images were obtained by both the high-voltage gating and the mechanical chopping methods. The experiment was performed during H-mode operation, and effective signals derived from single SR pulses (electron bunches of 5 mA, ∼50 ps) are picked up at a frequency of 5 kHz for both methods. The air-spindle-type chopper was employed for this experiment. While the images are considerably distorted in the voltage gating mode [Fig. 6(*a*) ▸], the spatial resolution of the image remains unchanged (about 200–300 nm under these experimental conditions, Fig. 6(*b*) ▸], compared with the case of usual static imaging (not shown) with the mechanical chopper. Moreover, the statistical efficiency is dramatically improved by using the mechanical chopper, which offers about six times the brightness of the electric gating mode [note that the two PEEM images in Figs. 6(*a*) and 6(*b*) ▸ were obtained with the same exposure time and are displayed on the same intensity scale]. This is due to the fact that the actual peak voltage applied to the channel plate does not reach the target value (600 V) with the electric gating method, since it is limited by the response speed of the conventional imaging plates. In the chopping mode, on the other hand, time-resolved images can be obtained with a DC voltage of 600 V applied to the imaging plate, since the unnecessary SR pulses/trains are eliminated mechanically.

The chopper systems developed in this study enable magnetic domain observations under the conditions of low-frequency probing (of the order of kHz) and narrow field of view (of the order of 10 µm), as shown in Figs. 6(*c*) and 6(*d*) ▸.

Both choppers work well in the present stage at the beamline. Comparing the two types of chopper:

(i) The air-spindle chopper has advantages in stability and cost. Stability here means smaller jitter and toughness against vibrations. Since the rotation axis is in air, trouble owing to the heat load and charging originating from X-rays can be prevented.

(ii) The magnetic-bearing chopper has advantages in vacuum and in higher rotation speed, in principle.

## 4. Summary   

We have developed two types of X-ray beam chopper suitable for pump-and-probe studies at the soft X-ray beamlines at SPring-8. One type uses an air spindle for the rotation axis, the other a magnetic bearing. Both types can be used under ultra-high-vacuum conditions. As a first demonstration, we have successfully combined the chopper with PEEM observation at BL25SU. The signal-to-noise ratio and resolution of the imaging results are drastically improved compared with those using the previous method of gating the high voltage applied to the channel-plate detector.

We plan to improve the magnetic-bearing-type design to achieve better ultra-high vacuum by altering some of the chopper components to use materials suitable for baking. The disc can be also exchanged for one designed for a repetition frequency of ∼209 kHz, which corresponds to the timing of one revolution of the SPring-8 storage ring. The systems are very useful for pump-and-probe time-resolved measurements in the soft X-ray region, and the designs could be adapted to be used at other synchrotron radiation facilities worldwide.
